# Supplementary material for: Sericin Ser3 Ectopic Expressed in Posterior Silk Gland Affects Hemolymph Immune Melanization Response via Reducing Melanin Synthesis in Silkworm
Source: Insects. 2023 Feb 28;14(3):245. doi: 10.3390/insects14030245 (PMC10051610; doi:10.3390/insects14030245)
Supplement: Supplementary file 1 [file insects-14-00245-s001.zip › insects-2216202-supplementary.pdf]

Supplemental Materials

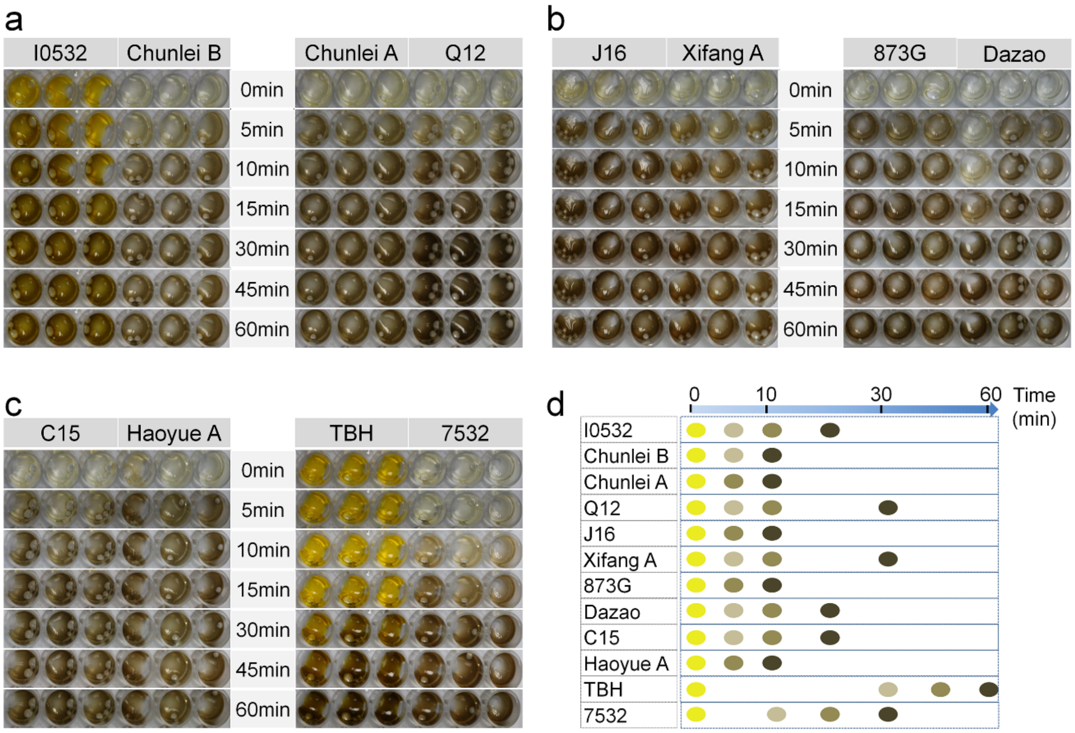

Figure S1. In vitro melanization of hemolymph of multiple silkworm varieties. (**a-c**) The melanization times of hemolymph from many varieties of silkworm. Silkworm varieties (strains) including I0532, Chunlei B, Chunlei A, Q12, J16, Xifang A, 873G, Dazao, C15, TBH and 7532. (**d**) Schematic diagram of hemolymph melanization times in vitro of multiple silkworm strains. According to the degree of melanization, there are four grades. The hemolymph just taken out is light yellow, and the beginning of oxidation and blackening is indicated by brown. The darker the color is, the deeper the degree of melanization is. Black means that the hemolymph is completely blackened.

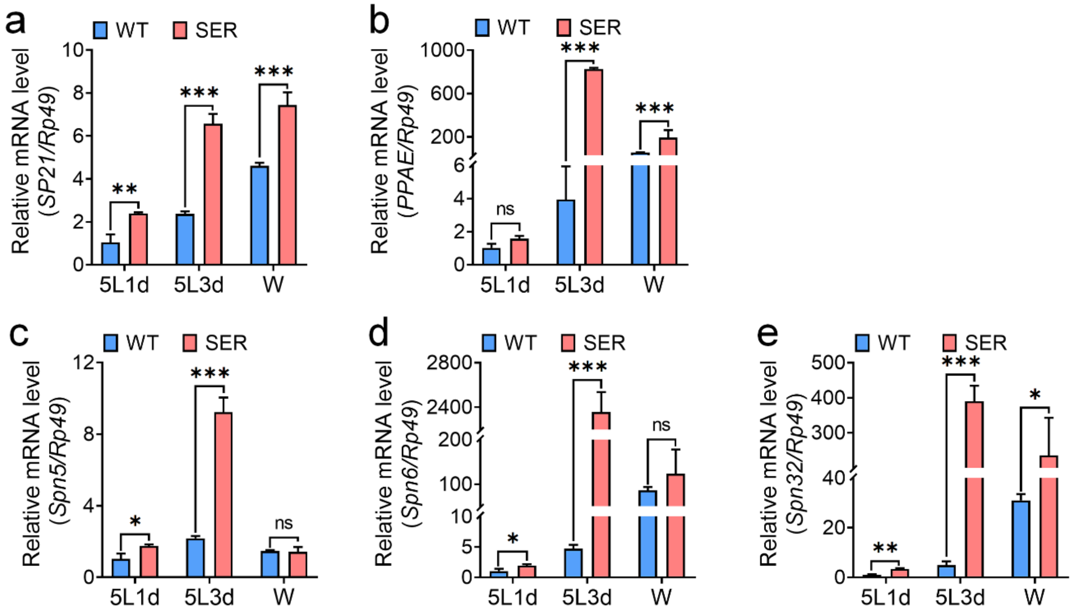

Figure S2. Changes in serine protease inhibitor gene transcription levels in fat body of transgenic silkworms. (**a-e**) qPCR was used to investigate the relative transcription level of genes. PPAE and SP21 are the positive regulatory genes that eventually activate PPO into PO; Spn5, Spn6 and Spn32 are the main member genes of the serine protease inhibitor family that are reversely regulated; and Rp49 was the internal reference gene. **p*<0.05; ***p*<0.01; ****p*<0.001; ns, no significant difference between the two groups. *n*=3 biological repeats.

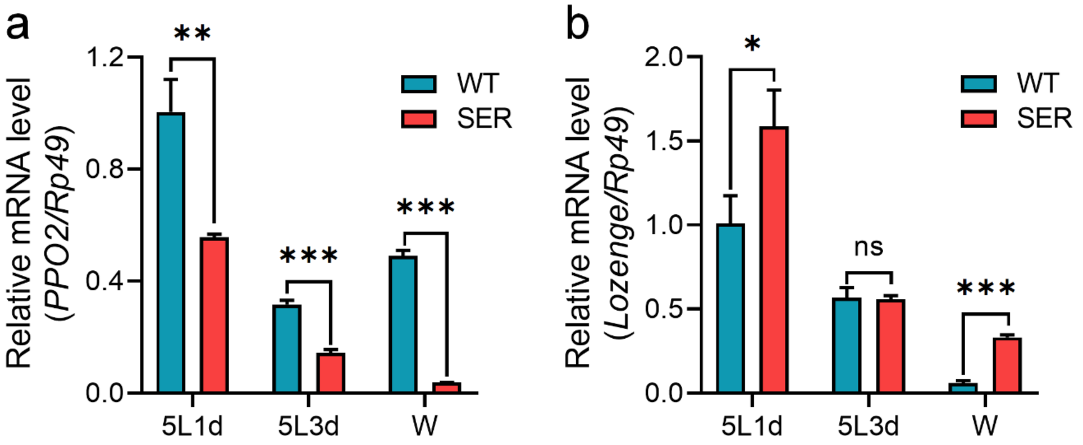

Figure S3. Changes in PPO2 and Lozenge gene transcription levels in hemolymph of transgenic silkworms. (**a**) PPO2, prophenoloxidase 2. (**b**) Lozenge. Rp49 was the internal reference gene. **p*<0.05; ***p*<0.01; ****p*<0.001; ns, no significant difference between the two groups. *n*=3 biological repeats.

Table S1. The primers used in the study

Gene name	Forward（5′→3′）	Reverse（5′→3′）	Gene ID
CAT	aatgtcggcggagatgtagacc	gcagcagcatccttgaggtgat	692456
DDC	ccaaccgctaactcgtat	ataaccacttccaactctgt	692675
SP21	cctatgattaccagtccaaca	ccttctcacacggatacac	100862834
PAH	cggtctactgtcgtcctt	ggctggtattctgtgatgg	101742825
PPAE	ggaggaatcttggatgtagg	ctgatacggacacgcaat	692368
PPO1	gagaatatgtccgtgatgct	ggttcctgctgatgatgg	692758
PPO2	gttactctgtggcactgat	gtggaacatctggtggaat	693073
Spn32	cggaatgccaatcaagaag	ggtcatcaagtctgaagtct	100272196
Spn5	agccagacttagccaact	ccgattccgtgacctcta	692688
Spn6	ttctactaccgccttactct	tctcaacgatgacttctctg	100126553
TH	ctgatataggcgatgacgat	agaagattcactagcagcat	100270767
TPX	actacggagtgctggacg	tcgggcttgatggtctt	733003
Cu/Zn-SOD	tcgtggtgatgttagcggaact	cgacatggcgtacagcagaact	692639
Mn-SOD	gattcgagttgccggtgcttct	gcttgtgcctgtgcgagtttct	692736
Moricin	gcaatgtctctggtgtca	gcgatattgatggctcttag	692365
CecropinA	ccaggtggaaactcttcaa	tttcgcttgccctatgac	693029
Lebocin	ggtatgttcgcagtgtca	ggtcaagtccagtatcaact	100146108
Attacin	gtgtagcgttgttgttgtt	aggttccatccgagttca	692555
Lozenge	tcacaggagatagcataactc	ggtccatcttgattgattgag	101747006
Rp49	cgtaaggtcctagttcacaa	cgtcactctgatgctgag	778453
